# Comparison of high resolution melting analysis, pyrosequencing, next generation sequencing and immunohistochemistry to conventional Sanger sequencing for the detection of p.V600E and non-p.V600E *BRAF* mutations

**DOI:** 10.1186/1471-2407-14-13

**Published:** 2014-01-10

**Authors:** Michaela Angelika Ihle, Jana Fassunke, Katharina König, Inga Grünewald, Max Schlaak, Nicole Kreuzberg, Lothar Tietze, Hans-Ulrich Schildhaus, Reinhard Büttner, Sabine Merkelbach-Bruse

**Affiliations:** 1 Institute of Pathology, University of Cologne, Medical Centre, Cologne, Germany; 2 Institute of Dermatology, University of Cologne, Medical Centre, Cologne, Germany; 3 Institute of Pathology, Ortenau-Hospital Lahr-Ettenheim, Lahr-Ettenheim, Germany; 4 Current address: Gerhard-Domagk-Institute of Pathology, University of Münster, Medical Centre, Münster, Germany; 5 Current address: Institute of Pathology, University Medical Centre, Göttingen, Germany

**Keywords:** HRM, cobas® *BRAF* V600 test, *therascreen*® BRAF pyro kit, Immunohistochemistry, Next generation sequencing, *BRAF* mutational analysis

## Abstract

**Background:**

The approval of vemurafenib in the US 2011 and in Europe 2012 improved the therapy of not resectable or metastatic melanoma. Patients carrying a substitution of valine to glutamic acid at codon 600 (p.V600E) or a substitution of valine to leucine (p.V600K) in *BRAF* show complete or partial response. Therefore, the precise identification of the underlying somatic mutations is essential. Herein, we evaluate the sensitivity, specificity and feasibility of six different methods for the detection of *BRAF* mutations.

**Methods:**

Samples harboring p.V600E mutations as well as rare mutations in *BRAF* exon 15 were compared to wildtype samples. DNA was extracted from formalin-fixed paraffin-embedded tissues by manual micro-dissection and automated extraction. *BRAF* mutational analysis was carried out by high resolution melting (HRM) analysis, pyrosequencing, allele specific PCR, next generation sequencing (NGS) and immunohistochemistry (IHC). All mutations were independently reassessed by Sanger sequencing. Due to the limited tumor tissue available different numbers of samples were analyzed with each method (82, 72, 60, 72, 49 and 82 respectively).

**Results:**

There was no difference in sensitivity between the HRM analysis and Sanger sequencing (98%). All mutations down to 6.6% allele frequency could be detected with 100% specificity. In contrast, pyrosequencing detected 100% of the mutations down to 5% allele frequency but exhibited only 90% specificity. The allele specific PCR failed to detect 16.3% of the mutations eligible for therapy with vemurafenib. NGS could analyze 100% of the cases with 100% specificity but exhibited 97.5% sensitivity. IHC showed once cross-reactivity with p.V600R but was a good amendment to HRM.

**Conclusion:**

Therefore, at present, a combination of HRM and IHC is recommended to increase sensitivity and specificity for routine diagnostic to fulfill the European requirements concerning vemurafenib therapy of melanoma patients.

## Background

The v-raf murine sarcoma viral oncogene homolog B1 (*BRAF*) is one of three *RAF* genes (rapidly accelerated fibrosarcoma A, B, C) localized on chromosome 7q34. This gene encodes a cytoplasmic serine-threonine protein kinase of the RAF family. RAF kinases are part of the mitogen-activated protein (MAP) kinase pathway involved in cell growth, survival and differentiation [1].

*BRAF* mutations play an important role in 40 – 70% of malignant melanomas, 45% of papillary thyroid cancers and 10% of colorectal cancers besides ovarian, breast and lung cancers [2-4].

According to the COSMIC database (Catalogue Of Somatic Mutations In Cancer, Dec 2013 [5]) 44% of the melanomas harbor *BRAF* mutations and 97.1% of these mutations are localized in codon 600 of the *BRAF* gene [6]. The most common variation is a substitution of valine to glutamic acid at codon 600 (c.1799 T > A, p.V600E or c.1799_1800TG > AA, p.V600E2; frequency 84.6%). Less common mutations are substitutions of valine to lysine (c.1798_1799GT > AA, p.V600K; frequency 7.7%), to arginine (c.1798_1799GT > AG, p.V600R; 1.0%), to leucin (c.1798G > A, p.V600M; 0.3%) or to aspartic acid (c.1799_1780TG > AT, p.V600D; 0.1%), mutations affecting codon 597 (p.L597; 0.5%), codon 594 (p.D594; 0.4%) and mutations in codon 601 resulting in the exchange of lysine to glutamic acid (c.1801A > G, p.K601E; 0.7%).

The approval of vemurafenib (PLX 4032, Roche Molecular Systems, Pleasanton, CA) in the US 2011 and in Europe 2012 improved the therapy of not resectable or metastatic melanoma. Vemurafenib exhibits an approximately 30-fold selectivity for p.V600E mutated compared to wildtype *BRAF* melanomas. In addition, patients carrying a p.V600K mutation included in the BRIM-3 study showed response to this inhibitor [7]. In a phase I trial, a 70% response rate to vemurafenib among 32 genotype selected metastatic melanoma patients was observed [8]. Recent *in vitro* and *in vivo* experiments indicate that vemurafenib might have an effect in patients with rare mutations in codon 600 of the *BRAF* gene [9-11] as for instance p.V600D or p.V600R [12,13]. Furthermore, dabrafenib (GSK2118436), another selective *BRAF* inhibitor [14] shows good clinical response rates not only for patients with p.V600E or p.V600K mutations but also in patients carrying a p.V600R, p.V600M or a double p.[V600E(;)V600M] mutation [15,16] giving new therapy options for melanoma patients with rare *BRAF* mutations.

The FDA approved vemurafenib with the cobas® *BRAF* V600 test (Roche) as companion diagnostic tool. The European Medicine Agency`s (EMA) Committee for Human Medicinal Products (CHMP) approved vemurafenib in February 2012 with two main differences to the FDA approval: a companion diagnostic test was not defined and treatment option is given for patients with melanomas carrying any mutation in codon 600 of the *BRAF* gene. Because a mutation in codon 600 determines eligibility for *BRAF* inhibitor treatment, several molecular screening methods have been developed. However, the level of validation and characterization of the performance features is not defined.

The aim of this study was to evaluate several parameters such as sensitivity and feasibility of different methods for the *BRAF* mutation analysis. Here, we compare the allele specific PCR done by the cobas® *BRAF* V600 test, the pyrosequencing using the *therascreen*® BRAF Pyro Kit (Qiagen), the high resolution melting (HRM) analysis, the immunohistochemistry (IHC), the next generation sequencing (NGS) approach and the bidirectional Sanger sequencing with regard to their sensitivity, specificity, costs, amount of work, feasibility and limitations. To our knowledge, this is the only study comparing these five PCR-based methods with IHC.

## Methods

### Samples

A total of 82 tumor samples were collected in the years 2010 until 2013 under approved ethical protocols complied with the Ethics Committee of the University of Cologne (Germany) and with informed consent from each patient. Of these, 63 samples were melanomas, 11 were lung adenocarcinomas and eight were colorectal carcinomas.

Tumors were diagnosed by an experienced pathologist (H. U. S., L. T.) and tumor content and pigmentation were defined. All samples were analyzed with Sanger sequencing as gold standard and the in-house method high resolution melting (HRM) analysis. The other methods were evaluated with a smaller number of samples due to the limited amount of tumor tissue available. Special attention was paid to the fact that each mutation type was once analyzed with each method. Overall 40 samples were at least analyzed with each of the six evaluated methods.

### DNA isolation

All samples were fixed in neutral-buffered formalin prior to paraffin embedding (FFPE-samples). On a haematoxylin-eosin stained slide tumor areas were selected by a pathologist (H.U.S.) and DNA was extracted from corresponding unstained 10 μm thick slides by manual micro-dissection. The DNA was isolated by automated extraction using the BioRobot M48 (Qiagen, Hilden, GER) following the manufacturer’s protocols. Quality and quantity of isolated DNA was assessed by agarose gel electrophoresis, by a Nanodrop 2000c spectrophotometer (PeqLab, Erlangen, GER) or in the case of next generation sequencing with the Qubit® Fluorometer (Life Technologies, Carlsbad, USA).

### High resolution melting analysis

High resolution melting (HRM) analysis was set up using 10 ng of genomic DNA, 3.5 mM MgCl_2_, 1× LightCycler 480 High Resolution Melting Master and 200 nM of each primer in a final reaction volume of 20 μl. Primer sequences were as follows: forward 5’- ATG CTT GCT CTG ATA GGA AAA TGA -3’ and reverse 5’- ATC CAG ACA ACT GTT CAA ACT -3’ with an annealing temperature of 59°C. Analyses were performed in duplicates using the LightCycler 480 platform (Roche Diagnostics, Mannheim, GER). Each run included a wildtype control and a mutant, p.V600E, control for normalization. Results were analyzed by Gene Scanning software with normalized, temperature-shifted melting curves displayed as difference plot. Samples showing a melting behavior differing from the wildtype control but not that of a mutant sample were considered as borderline samples. These samples were retested by direct Sanger sequencing of HRM products.

### Sanger sequencing

Sanger sequencing was performed on the same amplicons as used for HRM analysis. 5 μl of PCR products were purified with exonuclease I and Fast-AP (Thermo Fisher Scientific, Waltham, USA) for 15 min at 37°C and 15 min by 80°C. A sequencing reaction was set up with 1 μl of purified PCR products and the BigDye® Terminator v1.1 Cycle Sequencing Kit (Life Technologies) following the manufacturer’s instructions. The BigDye XTerminator® Purification Kit (Life Technologies) was used for the purification of the DNA sequencing reactions removing non-incorporated BigDye® terminators and salts. Solution was incubated for 30 min with agitation of 1800 rpm. Sequencing analyses were carried out on the eight capillary 3500 Genetic Analyzer (Life Technologies).

### Next generation sequencing

Targeted next generation sequencing (NGS) was performed on 72 FFPE samples. Isolated DNA (<0.5 – 97.6 ng/μl) was amplified with an in-house specified, customized Ion AmpliSeq Primer Pool. The panel comprises 102 amplicons of 14 different genes including exon 11 and 15 of the *BRAF* gene. PCR products were ligated to adapters and enriched for target regions using the Ion AmpliSeq Panel^TM^ Library kit according to manufacturer’s instructions (Life Technologies). The generated libraries were equimolar pooled for amplicon sequencing to a concentration of 20 nM of each sample to counterbalance differences in sample quality. Sequencing was performed on an Illumina MiSeq benchtop sequencer (Illumina, San Diego, USA). Results were visualized in the Integrative Genomics Viewer (IGV) [6] and manually analyzed. A 5% cutoff for variant calls was used and results were only interpreted if the coverage was >100.

### Pyrosequencing

Pyrosequencing was performed with the *therascreen*® BRAF Pyro Kit (Qiagen) detecting certain mutations in codon 600 of the *BRAF* gene according to manufacturer’s instructions. 1 μl of each isolated DNA was analyzed per run. Pyrosequencing was performed on the PyroMark Q24 platform (Qiagen) using the PyroMark Gold Q24 reagents. Pyrograms were generated with the PyroMark Q24 software (v. 2.0.6.) and data were analyzed manually or with a plug-in tool provided by Qiagen. Sequences surrounding the site of interest served as normalization and reference peaks for quantification and quality control. Dispensation order was as follows: 5’- GCT ACT GTA GCT AGT ACG AAC TCA-3’. Two different “sequence to analyze” were used: 5’- YAY TGT AGC TAG ACS AAA AYC ACC -3’ or 5’- CHC TGT AGC TAG ACS AAA ATY ACC -3’ for manual analysis. Samples with 5% mutated alleles or more were scored as mutation positive.

### Allele specific PCR

For the allele specific PCR the cobas® *BRAF* V600 test was utilized. DNA was isolated with the in-house method. Following the manufacturer’s instructions, 5 ng/μl DNA of each sample were analyzed on the cobas® z 480 system. If the concentration of the extracted DNA was too low, the maximum DNA volume of 25 μl was used. The results were displayed automatically as report by the cobas® z 480 software.

### Immunohistochemistry

Anti-*BRAF* p.V600E immunohistochemical staining was performed using the specific monoclonal mouse antibody VE1 (Spring Bioscience, Pleasanton, CA, USA; purchased from Zytomed Systems, Berlin, Germany). Dewaxing, heat induced epitope retrieval with citrate buffer, antibody incubation (dilution 1:50) and counterstaining were carried out on a BOND Max immunostainer by using Bond Epitope Retrieval Solution 1 and the Bond Polymer Refine Detection kit (Menarini, Berlin, Germany). Immunohistochemical staining was carried out within 2 weeks after cutting the 4 μm sections.

Staining results were scored from 0 to 3+ by a senior pathologist (H. U. S. or I. G.) blinded to the results of molecular analysis. The staining was considered as positive for p.V600E staining (2+ and 3+) when the majority of viable tumor cells showed clear cytoplasmic staining. Negative staining results were interpreted when there was no or only slight staining, staining of only single cells or of monocytes and macrophages (0 and 1+).

## Results and discussion

Precise identification of genomic alterations is essential for personalized therapy in cancer. Concerning melanoma, particularly patients carrying a mutation in codon 600 of the *BRAF* gene respond to vemurafenib [7,17]. As no companion diagnostic test for this drug is prescribed in Europe, we aimed at evaluating a sensitive and specific molecular method for *BRAF* mutation analysis by comparing high resolution melting (HRM) analysis, pyrosequencing (*therascreen*® BRAF Pyro Kit (Qiagen)), allele specific PCR (cobas® *BRAF* V600 test (Roche)), Sanger sequencing, next generation sequencing (NGS) and immunohistochemistry (IHC).

82 tumor samples (63 melanomas, 11 lung adenocarcinomas and eight colorectal carcinomas) evaluated during routine diagnostics from 2010 – 2013 and covering a wide range of different mutations as well as wildtype samples were subjected to analysis. Because of limited tumor tissue available we were not able to analyze all samples with each method but we paid attention to the fact that each mutation type was once analyzed with each method. At least, 40 samples were analyzed with all six evaluated methods. Lung adenocarcinomas as well as colorectal carcinomas were included into this study to get a broader spectrum of mutations. Hereby, the frequency of mutations other than p.V600E is significantly higher than in melanoma [18-20]. *BRAF* mutations were mainly found in codon 600, codon 469 and codon 594 of non-small-cell lung cancer (NSCLC) samples [21]. Furthermore, therapies targeting *BRAF* mutant tumors have recently been identified in NSCLC [22,23].

Tumor content and pigmentation was assessed by an experienced pathologist (H. U. S. or I. G.). The proportion of tumor cells ranged from 15 - 100% (Additional file 1) and pigmentation was scored as no (<5% of evaluated melanoma cells), low (5 - 49%) and high pigmentation (>50%).

### High resolution melting analysis and Sanger sequencing

**Figure 1:**
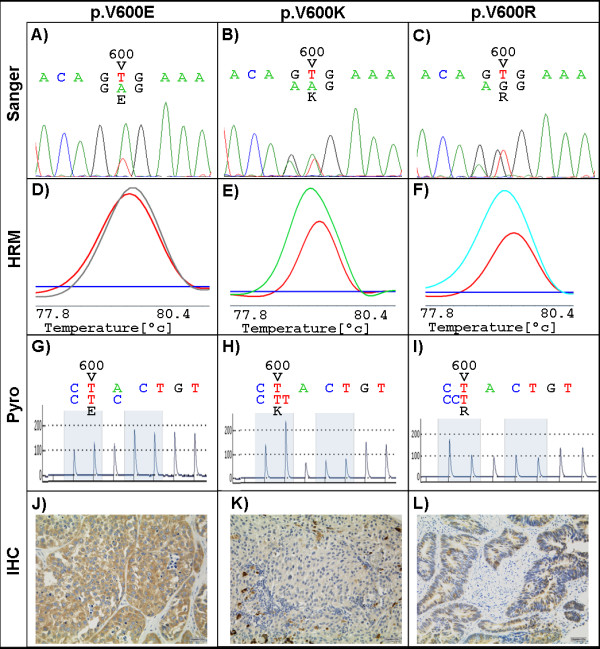
**Representative results for *BRAF *exon 15 mutation analysis.** Sanger sequencing **(A - C)**, high resolution melting (HRM) analysis **(D - F)**, pyrosequencing (Pyro) **(G - I)** and immunohistochemistry (IHC) **(J-L)** are compared: The first column shows exemplarily p.V600E mutations, the second p.V600K mutations and the third column p.V600R mutations. In HRM, normalized and temperature shifted difference plots showing wildtype control in blue and mutant control in red. HRM can distinguish between p.V600E (red) and p.V600K (green) and p.V600R (light blue). Pyrosequencing was performed in the reverse direction with the sequence to analyze 5’- YAY TGT AGC TAG ACS AAA AYC ACC -3’. All three mutations can be detected. Immunohistochemistry shows a strong staining for p.V600E **(J)** but is negative for p.V600K **(K)** in representative melanoma sample. Pigmentation has to be clearly distinguished from a positive p.V600E staining **(K)**. Cross reactivity was observed for p.V600R mutation **(L)** in a colorectal carcinoma sample. A: adenine, C: cytosine, G: guanine, T: thymine, V: valine, E: glutamic acid, K: lysine, R: arginine.

Using the high resolution melting (HRM) method and Sanger sequencing, 81 of 82 samples could be amplified (Additional file 1) and analyzed using the same PCR products. Cases were considered as mutated using HRM if a significant difference of the fluorescence level was detected that was outside the range of variation of the wildtype control. Samples in between wildtype control and a mutant melting behavior were considered as borderline results. All mutated as well as borderline samples were subjected to Sanger sequencing to determine the specific mutation type. The assay was set up with an amplicon of 163 base pairs and is therefore able to detect all hotspot mutations as well as rare mutations in the entire exon 15 (codon 582 to 620) of *BRAF* (specificity 100%). This is in concordance with the studies of Colomba et al. [24] and Tol et al. [25]. Figure 1 displays representative difference plots for *BRAF* p.V600E (D), p.V600K (E) and p.V600R (F) mutations. p.V600E mutation (control, shown in red) can be clearly distinguished from p.V600K mutation (shown in green) and p.V600R (shown in blue). Furthermore, electropherograms with common mutations in codon 600 of the *BRAF* gene analyzed by Sanger sequencing are shown: p.V600E (A), p.V600K (B) and p.V600R (C).

Only one sample with p.V600E mutation could neither be analyzed by Sanger sequencing nor by HRM because of amplification failure (1.2%). Others have shown, that melanin binds to and interferes with DNA polymerases resulting in invalid test results [26]. But this case had a tumor content of 80% and showed no pigmentation. Therefore, the failure of amplification of the 163 bp fragment for Sanger sequencing and HRM is rather due to the high degradation of FFPE-used material than to pigmentation. This high degradation of FFPE used material can also explain the higher Sanger sequencing failure rate described in other studies using a larger PCR product for analysis [24,27].

**Figure 2:**
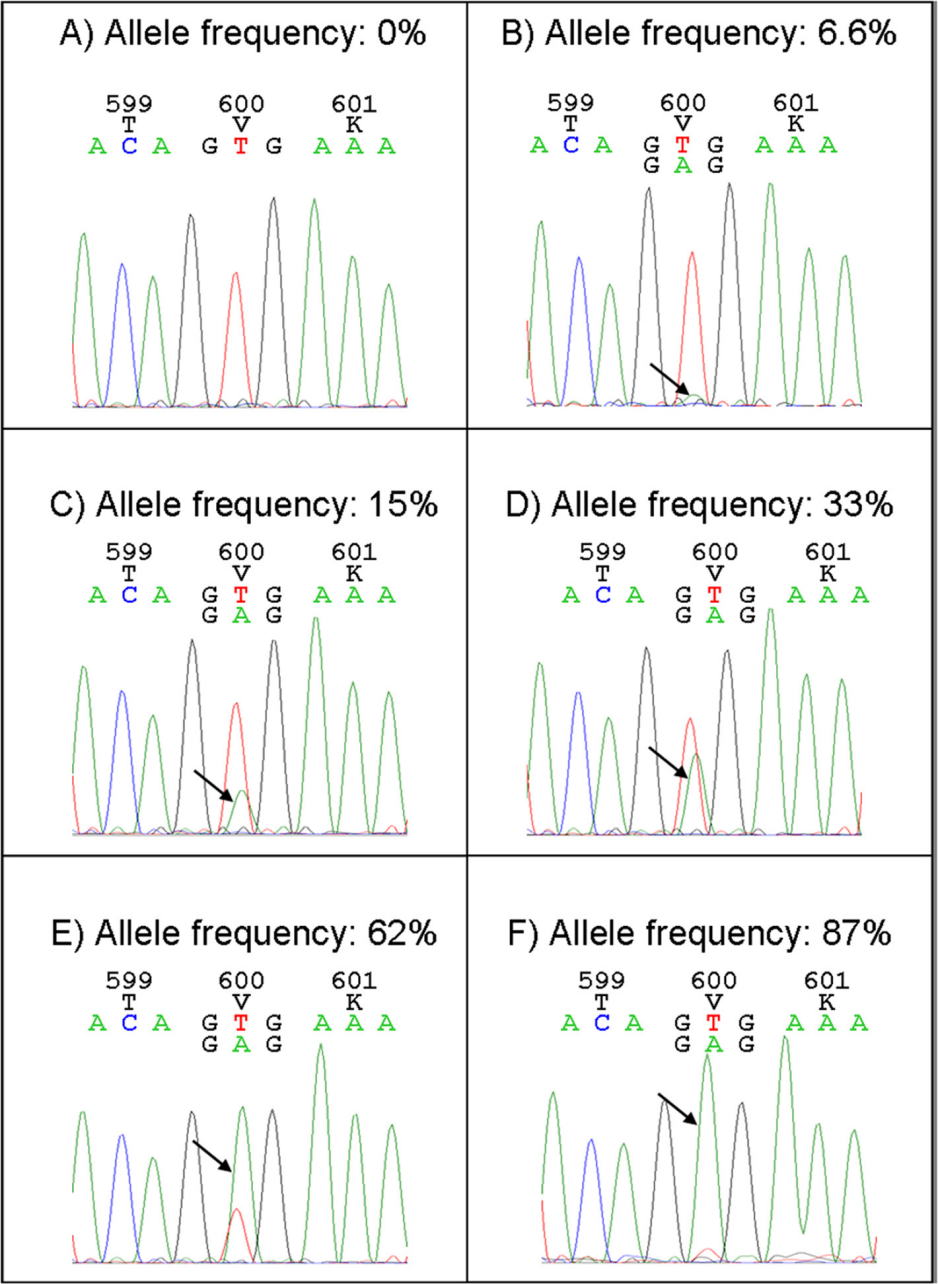
***BRAF *p.V600E mutation analysis of formalin-fixed paraffin-embedded tumor samples using Sanger sequencing.** Electropherograms showing that Sanger sequencing is not only able to detect mutations with high mutant allele frequency **(D - F)** but also mutations with rather low mutant allele frequency **(B and C)** compared to a wildtype sample **(A)**. Even 6.6% of *BRAF* p.V600E allele could be detected. A: adenine, C: cytosine, G: guanine, T: thymine, T: threonin, V: valine, K: lysine.

The sensitivity of Sanger sequencing is described in the literature as 20% mutated alleles in a background of wildtype alleles [28], but in the present study, we were able to detect 6.6% mutated alleles (Table 1, Additional file 1). Figure 2 shows six electropherograms of samples analyzed in this study with different allele frequencies according to next generation sequencing (A: 0%, B: 6.6%, C: 15%, D: 33%, E: 62% and F: 87%). B) shows that a sample with 6.6% allele frequency can be distinguished from a wildtype sample (A) and that an allele frequency of 15% can be clearly detected as p.V600E mutation using Sanger sequencing. HRM analysis has an even lower detection limit of 6.3% mutated alleles as reported by our group previously [29]. Carbonell et al. showed an even lower detection limit ranging from 1 – 5% [27]. This was also supported by Balic et al. [30] who showed that analyzing DNA methylation 1% methylated DNA in the background of unmethylated DNA could be reproducibly detected in fresh frozen as well as in FFPE samples.

**Table 1:** Summary of the properties of the evaluated methods

	**HRM**	**Pyro**	**Cobas**	**IHC**	**NGS**	**Sanger**
**CE mark**	No	No	Yes	No	No	No
**Limit of detection [%]**	6.6	5	7	5	2	6.6
**Detection of rare mutations**	Yes	Yes- downstream of codon 600	No- 4 different mutations	No- p.V600E	Yes	Yes
**Specificity [%]**	100	90	98.3	98	100	100
**Turnaround time**	1 day	2 days	1 day	1 day	3 - 5 days	2 - 3 days
**Costs**	Low	High	Medium	Low	Very high	Medium

HRM: high resolution melting analysis; Pyro: pyrosequencing with the *therascreen*® BRAF Pyro Kit; cobas: cobas® BRAF V600 test; IHC: immunohistochemistry; NGS: next generation sequencing on the MiSeq platform; Sanger: Sanger sequencing.

**Figure 3:**
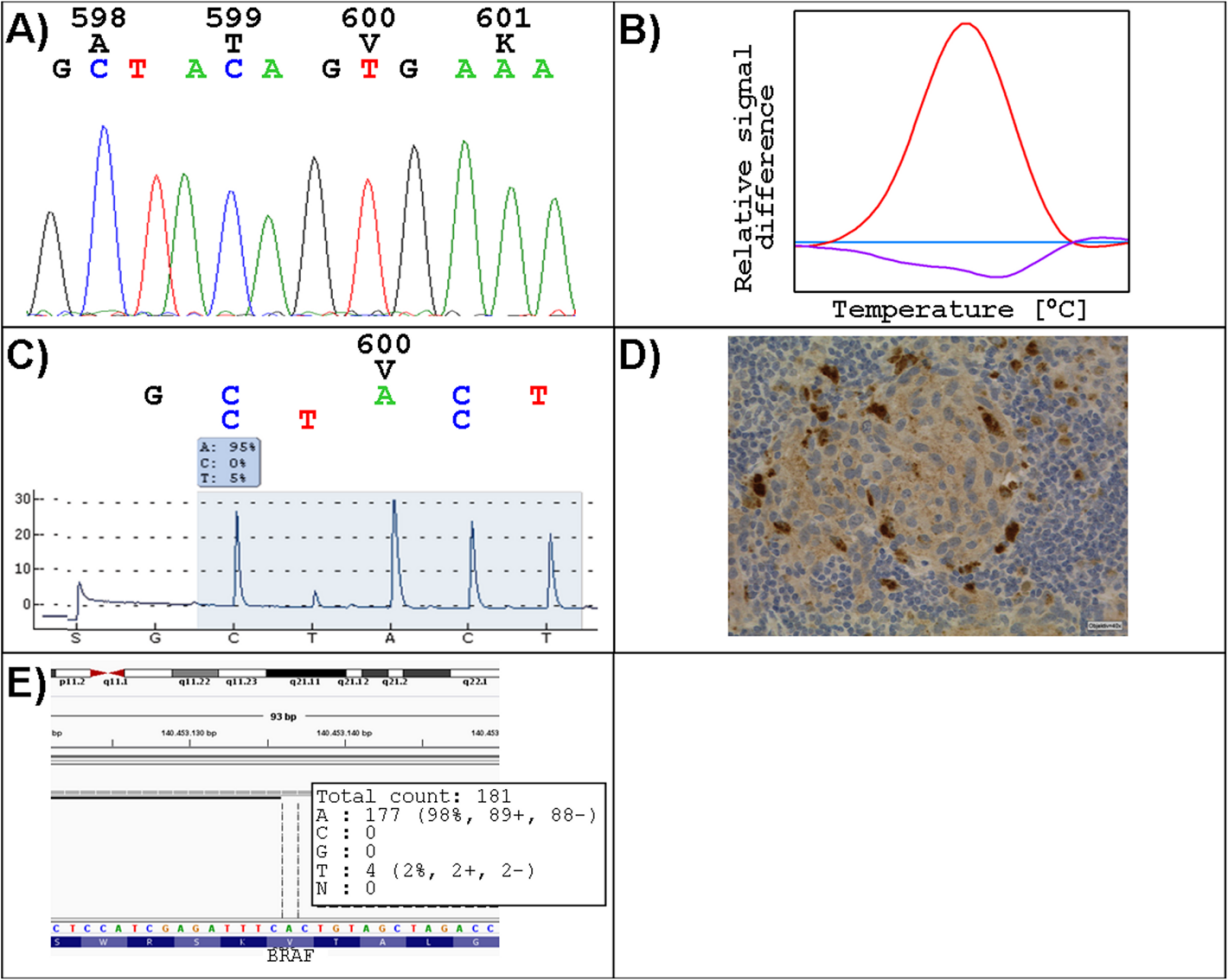
**Melanoma sample showing different results of the *BRAF *p.V600E mutational analysis (case 30).** Sanger sequencing as well as high resolution melting analysis show wildtype results (**A** and **B**, respectively). Pyrosequencing analysis resulted in a p.V600E mutation with a 5% mutant allele frequency having low relative fluorescent units of almost 30 **(C)**. This result is confirmed by immunohistochemistry **(D)**. Next generation sequencing resulted in a 2% mutant allele frequency with a coverage rate of 181 **(E)**.

99% of all mutations could be detected by HRM as well as by Sanger sequencing. Case 30 could be amplified and was wildtype using Sanger sequencing, HRM and the cobas® BRAF V600 test but exhibited a p.V600E *BRAF* mutation with an allele frequency between 5 and 2% using pyrosequencing and NGS. Immunohistochemistry was scored positively as 2+. Tumor content of this sample was 30% with a high pigmentation rate (case 30, Figure 3, Additional file 1). At least for Sanger sequencing, it was already reported that the tumor content may have influence on the sensitivity. Tol et al. demonstrated that the analysis of tumor samples containing more than 30% percent of tumor cells increased the sensitivity of Sanger sequencing in a cohort of 511 primary colorectal cancer samples [25].

Case 67, showing twice a borderline result in HRM, revealed a substitution from guanine to adenine in only one of four Sanger sequencing reactions. The cobas® *BRAF* V600 test was also negative. Therefore, this substitution was considered to be a fixation artifact and the case was classified as wildtype. A pitfall of all PCR based methods amplifying DNA from FFPE tissues is this occurrence of fixation artifacts [31,32]. To exclude such false-positive results, we highly recommend performing PCR amplification in duplicates prior to mutation analysis.

### Pyrosequencing

Pyrosequencing is a real-time sequencing by synthesis approach and allows the quantification of mutated alleles. The *therascreen*® BRAF Pyro Kit for exon 15 of *BRAF* is specific for mutations in codon 600 with a reported sensitivity for p.V600E of 2% mutated alleles in a background of wildtype alleles according to manufacturer’s reference. In addition, recent reports show that even rare mutations in codon 600 can be detected using pyrosequencing with a customer designed assay set up [24,33]. In our preselected cohort the minimum of mutated alleles detected with pyrosequencing was 5% (Table 1 and Additional file 1). This is in concordance with Tsiatis et al. showing as well a detection limit of 5% for pyrosequencing [34]. All 72 samples were successfully amplified and subjected to analysis. The PCR product has an estimated size of approximately 120 base pairs. Figure 1 shows representative pyrograms of *BRAF* p.V600E (G), p.V600K (H) and p.V600R (I) mutations. Only pyrograms showing peak heights of >/=30 relative fluorescent units (RLU) were evaluated. Result interpretation was once performed by visual inspection with different sequences to analyze and in some samples with a mutation in codon 600 using the provided plug-in tool (Qiagen).

Concerning the p.V600E mutation pyrosequencing showed a higher sensitivity than Sanger sequencing. Pyrosequencing detected the p.V600E mutation down to 5% mutated alleles in a background of wildtype alleles but with values for the relative fluorescent units close to our threshold of 30. Sanger sequencing, HRM and the cobas® *BRAF* V600 test failed to detect this mutation as described above. Immunohistochemistry was scored positively as 2+. Interestingly, NGS showed a 2% allele frequency for p.V600E in this case being under the cutoff defined for our study (case 30).

The *therascreen*® BRAF Pyro Kit sequences in the reverse direction starting at codon 600 of the *BRAF* gene. Therefore, mutations downstream of codon 600 will be identified either as false-negative wildtype samples or as false-positive p.V600E samples. According to COSMIC database (Catalogue of Somatic Mutations in Cancer, Dec 2013) 1.4% of mutations are consequently not detected [6]. In our study, three cases were falsely detected as p.V600E mutation showing once a p.K601E, once a p.V600K and once a p.[V600E(;)K601E] double mutation using Sanger sequencing and NGS (case 41, 36 and 25, respectively, Additional file 1). If these patients are treated with vemurafenib they may develop keratocanthoma and squamous-cell carcinoma caused by treatment with supposable limited clinical benefit [35,36].

Furthermore, as the read length of the pyrosequencing kit is optimized for the detection of p.V600E mutation, the peak height can be misinterpreted in the regions upstream of codon 600. Two cases that were wildtype using Sanger sequencing and NGS and showed borderline results in HRM exhibited a p.G596 mutation using pyrosequencing with a mutation frequency of 8 and 14% (case 29 and 39, Additional file 1) analyzed by the first sequence to analyze. A third case (case 31, Additional file 1) could not be amplified by Sanger sequencing and HRM but was p.G596R mutated using pyrosequencing (7% allele frequency). Computed analysis with a second sequence to analyze of all three samples showed no mutation in the pyrograms reinforcing the wildtype result of the other methods. A further case (case 32) exhibited a p.L597R mutation using Sanger sequencing and NGS (8% allele frequency) but the pyropgram showed a p.G596R mutation with an allele frequency of 28%. The sequence to analyze and the dispension order used are not designed to detect mutations in codon 597. The mutated nucleotide is therefore incorporated at the wrong position of the pyrogram resulting in an incorrect mutation calling.

Thus, pyrosequencing showed a specificity of 90% for the detection of all mutations in our preselected cohort (Table 1). According to the manufacturer the *therascreen*® BRAF Pyro Kit should only be used for mutations in codon 600 of the human *BRAF* gene. Regarding only the hotspot codon 600 pyrosequencing exhibited a specificity of 94.6%.

If using the *therascreen*® BRAF Pyro Kit for the detection of additional mutations the results should be critically considered especially concerning mutations in codon 597, 596 and 594 of the *BRAF* gene. This is in concordance with Gong et al., 2010 showing continuous loss of signal intensities using pyrosequencing when sequencing towards increased read length [37]. Moreover, the interpretation of complex mutations (e.g. double mutations) is prone to errors as only the ratio of the peak heights vary. In the study of Shen and Qin (2012) a p.V600K mutation was overlooked by visual inspection but was detected using pyrosequencing data analysis software [38]. Using software tools and a customer designed assay set-up can avoid such problems [39,40]. Besides, it allows the detection of a broader spectrum of mutations [40,41] and reduces the costs down to one-quarter.

### Allele specific PCR

The cobas® 4800 *BRAF* V600 test is the only CE-IVD marked test used in this study. The CE-IVD mark indicates that this test meets essential requirements regarding safety, health and environmental protection.

60 out of 82 tumor samples were analyzed with the cobas® *BRAF* V600 test (Table 1). All samples showed a valid result (100%). The allele specific PCR used in this test generates an amplicon of 116 base pairs containing codon 600 in exon 15 of the *BRAF* gene. Amplification curves are shown only for the mutant and the wildtype control but not for the samples analyzed and a non-template control is not provided. Data are analyzed when mutant and wildtype controls have a “valid” status. A report is generated automatically and results can be distinguished between “mutation detected” and “mutation not detected”. This test is specific for the p.V600E mutation with a reported sensitivity of ≥5% mutated alleles in a background of wildtype alleles [42]. Limit of detection in our preselected cohort was 7% mutant alleles in a background of wildtype alleles (Table 1).

36 of 37 p.V600E mutations were detected with the cobas® *BRAF* V600 test (97.2% sensitivity). One case with a borderline frequency of 5% of mutated alleles using pyrosequencing (Figure 3) could not be detected. But it should be taken into account that we extracted the DNA with our standard in-house method and not with the recommended kit. This may influence the test results. Furthermore, the marked area on the HE-stained slide contained many lymphocytes diluting the p.V600E alleles. Curry et al. showed an even lower limit of detection of 4.4% mutated alleles per 1.25 ng/μl on FFPE tissues for the p.V600E mutation [43]. In contrast, Lade-Keller et al. performed a dilution series of p.V600E mutated DNA followed by analysis on the cobas® 4800 *BRAF* V600 test. This test was not able to detect a p.V600E mutation on the dilution point that theoretically contained 10% mutant alleles [33].

Analysis have shown cross reactivity with p.V600E2 (>65% allele frequency), p.V600K (>35%) and p.V600D (>10%) but not with p.V600R mutation [43,44]. In our cohort, the cobas® BRAF V600 test showed cross reactivity five times in p.V600K mutated samples containing 59, 61, twice 62 and 64% of mutated alleles using pyrosequencing. One p.V600K mutation with a frequency of 57% that is above the described cross reactivity, was not detected by the cobas® 4800 *BRAF* V600 test.

Furthermore, several additional cases with a mutation frequency below the described limit of detection were missed in our study: case 9 showed a frequency of 6.6% for p.V600K, case 36 25% for the same mutation and case 24 an allele frequency of 46% for the p.V600E2 mutation (Additional file 1). Case 3, 33 and 38 showed a mutation frequency of 37, 42 and 39% for p.V600R mutation that can not be detected by this kit. This makes an overall failure rate of 13.3% in our preselected cohort and a failure rate of mutation located in codon 600 of 16.3%. Halait et al. even showed that the cobas® 4800 BRAF V600 test failed to detect 19% of the mutations occurring in codon 600 of the *BRAF* gene [45]. In the study of Curry et al. 82.3% of non-p.V600E mutations (p.V600K and p.V600R) were not detected having a tumor content range from 5 - 45% and 14% median mutant alleles. But recent studies showed that even patients with p.V600K, p.V600D and p.V600E2 mutation positive melanomas may benefit from therapy with vemurafenib [7,9,15,46,47]. Furthermore, patients with uncommon mutations as p.V600R and double mutations as e.g. p.[V600E(;)V600M] treated with dabrafenib showed response based on RECIST (Response Evaluation Criteria in Solid Tumors) criteria and regression of metastatic lesions [15].

As expected, all other mutations evaluated could not be detected by this method (Tables 1). 3.8% of all mutations detected in malignant melanomas are outside of codon 600 of the *BRAF* gene [5]. To date, there are 121 different missense mutations described for *BRAF*[6]. Especially the p.L597 mutation (frequency 0.5%) plays an important role as it seems to be associated with sensitivity to MEK inhibitor therapy with TAK-733 [48,49]. To conclude, in its present set-up, this test is not sufficient for the European approval of vemurafenib [50].

### Next generation sequencing

Next generation sequencing allows the sensitive and simultaneous detection of various mutations in different genes in a multiplex approach. 72 out of 82 cases were subjected to next generation sequencing (NGS). Coverage for *BRAF* exon 15 ranged from 352 to 20174 with a mean coverage of 5015.4. The coverage of the mutation site ranged from 118 to 12002 with a mean coverage of 1934.7. Rechsteiner et al. reported in a cohort of 81 colorectal carcinoma samples a coverage rate from 5139 to 17156 [51]. As the threshold of coverage was set to 100 all samples could be analyzed.

The whole mutational spectrum could be detected by NGS and all cases were analyzed successfully (Additional file 1). The cut-off value defined for reliable mutation detection was set as a frequency of 5% mutant alleles. With this cut-off all but one mutation were analyzed correctly (98.6% sensitivity). Case 30 (Figure 3) showed only a 2% mutant allele frequency in the Integrative Genomic Viewer [6]. Coverage rate using NGS was very low with 181 which may have influenced the results obtained. In the whole cohort the lowest frequency of mutant alleles detected with NGS was 7% (Additional file 1). This makes a specificity of 100% for NGS but a sensitivity of 98.6% (Table 1).

NGS is characterized by a high working load with a lot of hands-on time and high costs. These disadvantages are compensated by the multiplexing possibilities, the broad spectrum of mutations detected and the high sensitivity. Recent publications state that almost 75% of cancer gene variations may be missed by an approach analyzing only hotspot mutations [52].

The establishment of this rather new method for routine diagnostic is an ongoing process. The expertise in computational biology required to perform clinical NGS is significantly higher than for any other of the established methods. Especially, the result interpretation is challenging: Where to define the cut-off value for a reliable mutation, which spectrum of mutations to report, how to validate and to report the results, how to handle the massive data generated? Standardization and validation of the test procedure and the data interpretation, cost reduction and getting to know the pitfalls of this method are the challenges of the future.

### Immunohistochemistry

Immunohistochemistry (IHC) is characterized by a fast and cheap performance and allows the detection of even small amounts of tumor cells harboring the specific antigen. 49 of the 82 samples were subjected to immunohistochemistry. Staining was homogenous within the tumor cells as shown by other groups before [53]. Figure 1 shows representative immunohistochemical stainings of p.V600E mutation (J) and p.V600K mutation (K) both in a melanoma sample and p.V600R mutation (L) of a colorectal tumor.

Staining with the p.V600E specific monoclonal antibody detected all evaluated p.V600E mutations (100%). 22 of these p.V600E mutated samples were melanoma and two were colorectal tumors. Colomba et al. described in contrast a IHC failure rate of 7.2% in a cohort of 111 cases [24] due to equivocal staining.

Furthermore, case 25, showing a double mutation in codon 600 and codon 601 of the *BRAF* gene was scored negative in IHC (Additional file 1). This is in concordance with the study of Skorokhod et al. (2012) who could not detect the double mutation with the monoclonal VE1 antibody either [54]. Eight cases with non-p.V600E mutation were scored as 1+ and therefore negative in the IHC (p.V600K, p.L597R/S, p.E586K and p.D594N). Like for the cobas® 4800 *BRAF* V600 test this p.V600E specificity constitutes the major limitation of the IHC for routine diagnostics as a single test.

However, the IHC was not completely specific for the p.V600E mutation as cross reactivity was observed in one case with a p.V600R mutation that was scored as 2+ (Table 1). This is in contrary to most other studies reporting no cross reactivity with non-p.V600E mutations [54-56]. Only Heinzerling et al. (2013) found for one sample an immunohistochemical cross reactivity with p.V600K mutation [57]. Therefore, in our study this method is characterized by 100% sensitivity but only 98% specificity (Table 1). Long et al. showed a sensitivity of 97% and a specificity of 98% in a cohort of 100 samples [56].

**Figure 4:**
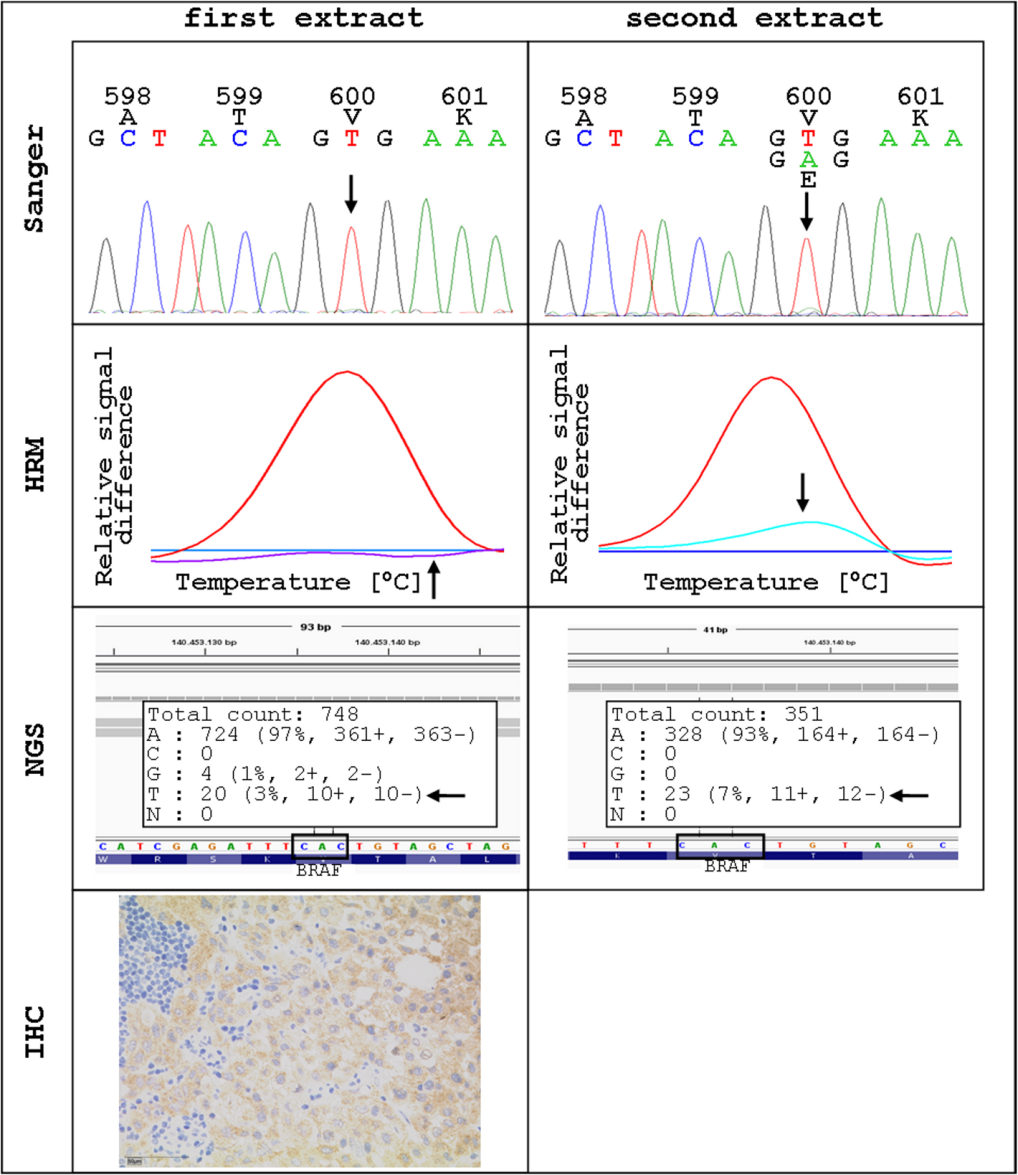
**Effects of different extracts on the same sample.** DNA from a melanoma sample (case 7) was extracted and showed wildtype results using Sanger, HRM and NGS (3% mutant allele frequency). In contrast, immunohistochemistry was positive. The second extract exhibited a p.V600E mutation with all evaluated methods. Sanger: Sanger sequencing, HRM: high resolution melting analysis, NGS: next generation sequencing, IHC: immunohistochemistry.

One case of our study highlighted the importance of immunohistochemical staining prior to DNA extraction for mutational analysis. Case 7 was wildtype using Sanger sequencing, HRM, and cobas® *BRAF* V600 test in the first extraction. NGS showed a p.V600E mutation with a 3% allele frequency being under our defined threshold. Sections for IHC were cut after the molecular analysis and results were positive with a score of 2+ by a senior pathologist (H. U. S.). Tumor content increased only slightly compared to the first H&E stained slide. Therefore, a second extraction was performed and analysis was repeated. The second extract showed a p.V600E mutation using Sanger sequencing, HRM, NGS as well as cobas® *BRAF* V600 test (Figure 4).

In general, Sanger sequencing needs 2 – 4 working days to produce a report. In contrast, HRM is time and cost saving and a major advantage is the prevention of contaminations as HRM is a close-tube process [58,59]. But it only serves as screening method not giving the exact mutational status. Advantages of pyrosequencing are that it is more sensitive than Sanger sequencing (5% versus 6.6% in our cohort) and the amount of work is lower compared to Sanger sequencing hence no clean up steps of the PCR-products is needed [34,40] but result interpretation is more prone to errors. The cobas® 4800 *BRAF* V600 test is characterized by an easy and fast performance with a low amount of work. Costs are medium compared to the other evaluated methods (Table 1). Immunohistochemistry (IHC) is characterized by a fast and cheap performance and allows the detection of even small amounts of tumor cells harboring the specific antigen but is limited to the detection of p.V600E mutations. NGS should be carefully validated to implement this method into routine diagnostics. At the moment it is only financially feasible when the full capacity of the device is used.

## Conclusion

To conclude, this is so far the only study comparing these five molecular methods with immunohistochemistry. We could show that Sanger sequencing as a well established tool is a reliable method for *BRAF* mutation analysis with a limit of detection of 6.6%. However, this method has to be replaced by faster and more cost effective methods. The cobas® 4800 *BRAF* V600 test has limited utilization as it detects only p.V600E mutations losing 16.3% of patients eligible for a therapy with vemurafenib. The pyrosequencing approach showed in fact the highest sensitivity in our preselected cohort with a limit of detection of 5% mutant alleles but exhibited the lowest specificity with 90% and is prone to errors without using customer designed set-up. In their present set-up, the cobas® 4800 *BRAF* V600 test as well as the *therascreen*® BRAF Pyro Kit are therefore not sufficient for the European approval of vemurafenib because there is a therapeutic option for melanoma patients with any mutation in codon 600 of the *BRAF* gene [50]. Therefore, we suggest a combination of VE1 antibody staining and HRM for p.V600E mutation analysis combining the lowest detection limit with a fast, reliable method with 100% sensitivity for routine diagnostics at the moment.

In the near future and with growing experiences, it is an inevitable fact that NGS will replace all established methods for molecular diagnostics. This is based on the high sensitivity and multiplexing options of this method allowing to generate a molecular profile of each tumor sample analyzed.

## Competing interests

The authors declare that they have no competing interests.

## Authors’ contributions

MAI carried out the molecular analysis and drafted the manuscript. JF established and performed the pyrosequencing analysis whereas KK established the NGS approach and made substantial contributions to the NGS analysis. MS, NK, LT and HUS performed pathological review of clinical material. HUS further analyzed IHC samples and contributed to the manuscript writing. IG analyzed IHC samples as a second reviewer. RB revised the manuscript critically for important intellectual content. SMB. supervised experiments, wrote the paper and revised critically the final manuscript. All authors read and approved the final manuscript.

## Pre-publication history

The pre-publication history for this paper can be accessed here:

http://www.biomedcentral.com/1471-2407/14/13/prepub

## Supplementary Material

Additional file 1 **Summary of *BRAF* mutation detection using five different molecular methods compared to immunohistochemistry.** Click here for file
