# Supplementary material for: Comparison of high resolution melting analysis, pyrosequencing, next generation sequencing and immunohistochemistry to conventional Sanger sequencing for the detection of p.V600E and non-p.V600E BRAF mutations
Source: BMC Cancer. 2014 Jan 10;14:13. doi: 10.1186/1471-2407-14-13 (PMC3893431; doi:10.1186/1471-2407-14-13)
Supplement: Additional file 1 — Summary of BRAF mutation detection using five different molecular methods compared to immunohistochemistry. [file 1471-2407-14-13-S1.doc]

# Additional file

Additional table 1: Summary of *BRAF* mutation detection using five different molecular methods compared to immunohistochemistry

| **Case No.** | **Tumor content [%]** | **Melanin content** | **Entity** | **Sanger sequencing** | **HRM** | **Pyrosequencing** | **NGS** | **Cobas** | **IHC** |
| --- | --- | --- | --- | --- | --- | --- | --- | --- | --- |
| 1 | ND | ND | Melanoma | c.1798_1799GT>AA, p.V600K | mut | ND | ND | ND | ND |
| 2 | 80 | Negative | Melanoma | c.1798_1799GT>AA, p.V600K | mut | c.1798_1799GT>AA, p.V600K (64%) | c.1798_1799GT>AA, p.V600K (62%) | detected | ND |
| 3 | 80 | Negative | Melanoma | c.1798_1799GT>AG, p.V600R | mut | c.1798_1799GT>AG, p.V600R (37%) | ND | not detected | ND |
| 4 | 80 | Negative | Melanoma | c.1799T>A, p.V600E | mut | c.1799T>A, p.V600E (36%) | c.1799T>A, p.V600E (39%) | detected | ND |
| 5 | 60 | Negative | Melanoma | c.1799T>A, p.V600E | mut | c.1799T>A, p.V600E (63%) | c.1799T>A, p.V600E (62%) | detected | ND |
| 6 | 90 | Low | Melanoma | c.1799T>A, p.V600E | mut | c.1799T>A, p.V600E (26%) | c.1799T>A, p.V600E (33%) | detected | ND |
| 7 | 40 | Negative | Melanoma | 1. wt  2. c.1799T>A, p.V600E | 1. wt  2. mut | 1. wt  2. c.1799T>A, p.V600E (9%) | 1. wt  2. c.1799T>A, p.V600E (7%) | 1. not detected  2. detected | 2+ |
| 8 | 20 | Negative | Melanoma | c.1799T>A, p.V600E | mut | c.1799T>A, p.V600E (15%) | c.1799T>A, p.V600E (21%) | detected | 2+ |
| 9 | 90 | Negative | Melanoma | c.1798_1799GT>AA, p.V600K | mut | c.1798_1799GT>AA, p.V600K (6.6%) | ND | not detected | ND |
| 10 | 10 | Negative | Melanoma | c.1799T>A, p.V600E | mut | ND | ND | detected | ND |
| 11 | 80 | Negative | Melanoma | c.1799T>A, p.V600E | mut | c.1799T>A, p.V600E (22%) | c.1799T>A, p.V600E (20%) | detected | ND |
| 12 | 90 | High | Melanoma | c.1799T>A, p.V600E | mut | c.1799T>A, p.V600E (50%) | c.1799T>A, p.V600E (50%) | detected | ND |
| 13 | 90 | Negative | Melanoma | wt | wt | wt | wt | not detected | negative |
| 14 | 90 | Low | Melanoma | c.1799T>A, p.V600E | mut | c.1799T>A, p.V600E (27%) | c.1799T>A, p.V600E (31%) | detected | ND |
| 15 | 60 | High | Melanoma | c.1799T>A, p.V600E | mut | c.1799T>A, p.V600E (25%) | c.1799T>A, p.V600E (23%) | detected | ND |
| 16 | 100 | Negative | Melanoma | wt | wt | wt | ND | not detected | ND |
| 17 | ND | ND | Melanoma | c.1798_1799GT>AA, p.V600K | mut | c.1798_1799GT>AA, p.V600K (62%) | c.1798_1799GT>AA, p.V600K (60%) | detected | ND |
| 18 | 80 | High | Melanoma | c.1799T>A, p.V600E | mut | c.1799T>A, p.V600E (49%) | c.1799T>A, p.V600E (36%) | detected | 2+ |
| 19 | 80 | Low | Melanoma | c.1798_1799GT>AA, p.V600K | mut | c.1798_1799GT>AA, p.V600K (27%) | ND | ND | 1+ |
| 20 | 60 | Negative | Melanoma | c.1798_1799GT>AG, p.V600R | mut | c.1798_1799GT>AG, p.V600R (36%) | c.1798_1799GT>AG, p.V600R (35%) | ND | negative |
| 21 | 80 | Negative | Melanoma | c.1790R>G, p.L597R | mut | ND | ND | ND | 1+ |
| 22 | 60 | Negative | Melanoma | c.1799_1780TG>AA, p.V600E2 | mut | c.1799_1780TG>AA, p.V600E2 (65%) | c.1799_1780TG>AA, p.V600E2 (50%) | ND | 3+ |
| 23 | 80 | Negative | Melanoma | c.1799T>A, p.V600E | wt* | c.1799T>A, p.V600E (49%) | c.1799T>A, p.V600E (87%) | detected | 2+ |
| **Case No.** | **Tumor content [%]** | **Melanin content** | **Entity** | **Sanger sequencing** | **HRM** | **Pyrosequencing** | **NGS** | **Cobas** | **IHC** |
| 24 | ND | ND | Melanoma | c.1799_1780TG>AA, p.V600E2 | mut | c.1799_1780TG>AA, p.V600E2 (46%) | c.1799_1780TG>AA, p.V600E2 (18%) | not detected | ND |
| 25 | 80 | Negative | Melanoma | c.[1799T>A(;)1801A>G], p.[V600E(;)K601E] | mut | c.1799T>A, p.V600E (7%) | c.[1799T>A(;)1801A>G], p.[V600E(;)K601E] (66%) | ND | negative |
| 26 | 50 | Negative | Melanoma | c.1798_1799GT>AA, p.V600K | mut | c.1798_1799GT>AA, p.V600K (20%) | c.1798_1799GT>AA, p.V600K (23%) | ND | negative |
| 27 | 70 | Low | Melanoma | c.1799_1800TG>AA, p.V600E2 | mut | c.1799_1800TG>AA, p.V600E2 (57%) | c.1799_1800TG>AA, p.V600E2 (66%) | ND | 2+ |
| 28 | ND | ND | Melanoma | c.1750C>T, p.L584F | mut | ND | c.1750C>T, p.L584F (28%) | ND | ND |
| 29 | ND | ND | Melanoma | wt | wt* | c.1786G>C, p.G596R (8%) | wt | ND | negative |
| 30 | 30 | High | Melanoma | wt | wt | c.1799T>A, p.V600E (5%) | wt | not detectecd | 2+ |
| 31 | 80 | Negative | Melanoma | invalid | invalid | c.1786G>C, p.G596R (7%) | wt | ND | ND |
| 32 | 20 | Negative | Melanoma | c.1790T>G, p.L597R | mut | c.1786G>C, p.G596R (28%) | c.1790T>G, p.L597R (8%) | not detected | 1+ |
| 33 | 70 | High | Melanoma | c.1798_1799GT>AG, p.V600R | mut | c.1798_1799GT>AG, p.V600R (42%) | c.1798_1799GT>AG, p.V600R (53%) | not detected | negative |
| 34 | 80 | Low | Melanoma | c.1798_1799GT>AA, p.V600K | mut | c.1798_1799GT>AA, p.V600K (59%) | c.1798_1799GT>AA, p.V600K (40%) | detected | negative |
| 35 | 80 | Negative | Melanoma | c.1799T>A, p.V600E | mut | c.1799T>A, p.V600E (68%) | c.1799T>A, p.V600E (67%) | detected | 3+ |
| 36 | 50 | High | Melanoma | c.1798_1799GT>AA, p.V600K | mut | c.1799G>A, p.V600E (52%) | c.1798_1799GT>AA, p.V600K (25%) | not detected | 1+ |
| 37 | 50 | Negative | Melanoma | c.1798_1799GT>AA, p.V600K | mut | c.1798_1799GT>AA, p.V600K (62%) | c.1798_1799GT>AA, p.V600K (56%) | detected | negative |
| 38 | 80 | Negative | Melanoma | c.1799T>A, p.V600E | mut | c.1799T>A, p.V600E (68%) | c.1799T>A, p.V600E (64%) | detected | 3+ |
| 39 | 40 | Negative | Melanoma | c.1798_1799GT>AA, p.V600K | mut | c.1798_1799GT>AA, p.V600K (63%) | c.1798_1799GT>AA, p.V600K (57%) | not detected | 1+ |
| 40 | 80 | Negative | Melanoma | c.1799T>A, .V600E | mut | c.1799T>A, .V600E (48%) | c.1799T>A, p.V600E (49%) | detected | 3+ |
| 41 | 60 | Low | Melanoma | c.1799T>A, p.V600E | mut | c.1799T>A, .V600E (40%) | c.1799T>A, .V600E (41%) | detected | 3+ |
| 42 | 50 | Low | Melanoma | c.1799T>A, p.V600E | wt* | c.1799T>A, .V600E (11%) | c.1799T>A, p.V600E (13%) | detected | 3+ |
| 43 | 15 | Negative | Melanoma | c.1799T>A, p.V600E | mut | c.1799T>A, .V600E (9%) | c.1799T>A, p.V600E (10%) | detected | 2+ |
| 44 | 80 | Negative | Melanoma | c.1799T>A, p.V600E | mut | c.1799T>A, .V600E (65%) | c.1799T>A, p.V600E (71%) | detected | 3+ |
| 45 | 90 | Negative | Melanoma | c. 1799T>A, p.V600E | mut | c.1799T>A, .V600E (70%) | c.1799T>A, p.V600E (71%) | detected | 3+ |
| 46 | 70 | Negative | Melanoma | c.1799T>A, p.V600E | mut | c.1799T>A, .V600E (74%) | c.1799T>A, p.V600E (74%) | detected | 3+ |
| 47 | 80 | Negative | Melanoma | c.1799T>A, p.V600E | mut | c.1799T>A, .V600E (70%) | c.1799T>A, p.V600E (74%) | detected | 3+ |
| 48 | 80 | Negative | Melanoma | c.1799T>A, p.V600E | mut | c.1799T>A, .V600E (71%) | c.1799T>A, p.V600E (66%) | detected |  |
| 49 | 70 | Negative | Melanoma | c.1799T>A, p.V600E | mut | c.1799T>A, .V600E (60%) | c.1799T>A, p.V600E (63%) | detected | 3+ |
| 50 | ND | ND | Melanoma | c.1799T>A, p.V600E | mut | c.1799T>A, .V600E (39%) | c.1799T>A, p.V600E (61%) | detected |  |
| **Case No.** | **Tumor content [%]** | **Melanin content** | **Entity** | **Sanger sequencing** | **HRM** | **Pyrosequencing** | **NGS** | **Cobas** | **IHC** |
| 51 | 80 | Negative | Melanoma | c.1799T>A, p.V600E | wt* | c.1799T>A, .V600E (44%) | c.1799T>A, p.V600E (71%) | detected | 2+ |
| 52 | 50 | Low | Melanoma | c.1799T>A, p.V600E | mut | c.1799T>A, .V600E (22%) | c.1799T>A, p.V600E (24%) | detected | 3+ |
| 53 | 80 | Negative | Melanoma | c.1799T>A, p.V600E | mut | c.1799T>A, .V600E (43%) | c.1799T>A, p.V600E (49%) | detected | 3+ |
| 54 | 60 | Negative | Melanoma | c.1799T>A, p.V600E | mut | c.1799T>A, .V600E (29%) | c.1799T>A, p.V600E (25%) | detected | 2+ |
| 55 | ND | ND | Melanoma | c.1799T>A, p.V600E | mut | c.1799T>A, .V600E (26%) | c.1799T>A, p.V600E (25%) | detected |  |
| 56 | 50 | Negative | Melanoma | c.1799T>A, p.V600E | mut | c.1799T>A, .V600E (7%) | c.1799T>A, p.V600E (12%) | detected | 3+ |
| 57 | 80 | Negative | Melanoma | c.1799T>A, p.V600E | mut | c.1799T>A, .V600E (37%) | c.1799T>A, p.V600E (57%) | detected | 3+ |
| 58 | 80 | Negative | Melanoma | c.1799T>A, p.V600E | mut | c.1799T>A, .V600E (37%) | c.1799T>A, p.V600E (45%) | detected | 2+ |
| 59 | 70 | Negative | Melanoma | c.1799T>A, p.V600E | mut | c.1799T>A, .V600E (38%) | c.1799T>A, p.V600E (67%) | detected | 3+ |
| 60 | 60 | Negative | Melanoma | c.1789_1790CT>TC, p.L597S | mut | c.1786G>C, p.G596R (11%) | c.1789_1790CT>TC, p.L597S (39%) | not detected | 1+ |
| 61 |  |  | Melanoma | c.1798_1799GT>AA, p.V600K | mut | c.1798_1799GT>AA, p.V600K (61%) | c.1798_1799GT>AA, p.V600K (49%) | detected |  |
| 62 | 60 | Low | Melanoma | c.1756G>A, p.E586K | mut | c.1786G>C, p.G596R (10%) | c.1756G>A, p.E586K (17%) | not detected | 1+ |
| 63 | 30 | Negative | Melanoma | c.1799T>A, p.V600E | mut | c.1799T>A, .V600E (31%) | c.1799T>A, p.V600E (34%) | detected | 2+ |
| 64 | 80 | ND | Colorectal tumor | c.1780G>A, p.D594N | mut | c.1780G>A, p.D594N (86%) | c.1780G>A, p.D594N (31%) | not detected | 1+ |
| 65 | 60 | ND | Colorectal tumor | c.1799T>A, p.V600E | mut | c.1799T>A, p.V600E (37%) | c.1799T>A, p.V600E (34%) | detected | 2+ |
| 66 | 60 | ND | Colorectal tumor | c.1799T>A, p.V600E | mut | c.1799T>A, p.V600E (17%) | c.1799T>A, p.V600E (15%) | detected | 3+ |
| 67 | 30 | ND | Colorectal tumor | wt | wt* | N/A | wt | not detected | negative |
| 68 | 40 | ND | Colorectal tumor | wt | wt | N/A | wt | not detected | ND |
| 69 | ND | ND | Colorectal tumor | wt | wt | wt | wt | not detected | ND |
| 70 | ND | ND | Colorectal tumor | c.1798_1799GT>AG, p.V600R | mut | c.1798_1799GT>AG, p.V600R (39%) | c.1798_1799GT>AG, p.V600R (42%) | not detected | 2+ |
| 71 | 30 | ND | Colorectal tumor | wt | wt* | p.G596 (14%) | wt | ND | ND |
| 72 | 70 | ND | Adenocarcinoma of the lung | wt | wt | ND | ND | ND | ND |
| 73 | 50 | ND | Adenocarcinoma of the lung | c.1801A>G, p.K601E | mut | c.1799T>A, p.V600E (88%) | c.1801A>G, p.K601E (27%) | not detected | negative |
| 74 | ND | ND | Adenocarcinoma of the lung | wt | wt | wt | wt | ND | ND |
| 75 | 40 | ND | Adenocarcinoma of the lung | Substitution in Intron 14 | wt* | wt | wt | ND | ND |
| 76 | 80 | ND | Adenocarcinoma of the lung | c.1781A>G, p.D594G | mut | c.1781A>G, p.D594G (28%) | c.1781A>G, p.D594G (19%) | ND | ND |
| 77 | ND | ND | Adenocarcinoma of the lung | c.1780G>A, p.D594N | mut | c.1780G>A, p.D594N (11%) | c.1780G>A, p.D594N (19%) | ND | ND |
| 78 | ND | ND | Adenocarcinoma of the lung | c.1786G>C, p.G596R | mut | c.1786G>C, p.G596R (28%) | c.1786G>C, p.G596R (20%) | ND | negative |
| **Case No.** | **Tumor content [%]** | **Melanin content** | **Entity** | **Sanger sequencing** | **HRM** | **Pyrosequencing** | **NGS** | **Cobas** | **IHC** |
| 79 | ND | ND | Adenocarcinoma of the lung | c.1782T>A, p.D594E | mut | ND | c.1782T>A, p.D594E (11) | ND | ND |
| 80 | ND | ND | Adenocarcinoma of the lung | c.1782T>A, p.D594E | mut | ND | ND | ND | ND |
| 81 | 40 | ND | Adenocarcinoma of the lung | c.1801A>G, p.K601E | mut | ND | c.1801A>G, p.K601E (52%) | ND | ND |
| 82 | 70 | ND | Adenocarcinoma of the lung | c.1786G>C, p.G596R | mut | c.1786G>C, p.G596R (20%) | ND | ND | ND |

invalid: no result was obtained by this method (no PCR product); ND: not done; *: borderline results; wt: wildtype; mut: mutation detected; HRM: high resolution melting analysis; NGS: next generation sequencing; IHC: immunohistochemistry; 1.: first extract; 2.: second extract
